# Residual risks and evolving atherosclerotic plaques

**DOI:** 10.1007/s11010-023-04689-0

**Published:** 2023-03-10

**Authors:** Sunil K. Noothi, Mohamed Radwan Ahmed, Devendra K. Agrawal

**Affiliations:** Department of Translational Research, Western University of Health Sciences, 309 E. Second Street, Pomona, CA USA

**Keywords:** Acute cardiac event, Atherosclerosis, Fibrous cap, Inflammation, Residual risk, Stable Plaque, Unstable plaque, Vulnerability

## Abstract

Atherosclerotic disease of the coronary and carotid arteries is the primary global cause of significant mortality and morbidity. The chronic occlusive diseases have changed the epidemiological landscape of health problems both in developed and the developing countries. Despite the enormous benefit of advanced revascularization techniques, use of statins, and successful attempts of targeting modifiable risk factors, like smoking and exercise in the last four decades, there is still a definite “residual risk” in the population, as evidenced by many prevalent and new cases every year. Here, we highlight the burden of the atherosclerotic diseases and provide substantial clinical evidence of the residual risks in these diseases despite advanced management settings, with emphasis on strokes and cardiovascular risks. We critically discussed the concepts and potential underlying mechanisms of the evolving atherosclerotic plaques in the coronary and carotid arteries. This has changed our understanding of the plaque biology, the progression of unstable vs stable plaques, and the evolution of plaque prior to the occurrence of a major adverse atherothrombotic event. This has been facilitated using intravascular ultrasound, optical coherence tomography, and near-infrared spectroscopy in the clinical settings to achieve surrogate end points. These techniques are now providing exquisite information on plaque size, composition, lipid volume, fibrous cap thickness and other features that were previously not possible with conventional angiography.

## Introduction

In recent years, vascular diseases have seen an epidemiological transition due to predominance of atherosclerotic occlusion of coronary and carotid artery and plaque rupture leading to myocardial infarction (MI) and stroke [1]. Even though the age-standardized rates of the prevalence and incidence of ischemic heart disease (IHD, the major contributor of the atherosclerosis burden) have decreased in last three decades, the absolute number of the same has been increasing because of population growth, ageing and increased lifespan all over the world. In 2017, IHD affected 126.5 million (95% uncertainty interval with a range of 118.6–134.7) people worldwide, an increase of 74.9% (95% uncertainty interval with a range of 71.8–78.6) compared with the 8.9 million (95% uncertainty interval with a range of 8.8–9.1) deaths in 1990. The incident cases of IHD were 10.6 million (95% uncertainty interval with a range of 9.6–11.8) in 2017, an increase of 51.8% compared with that in 1990. The global burden of IHD as measured by the absolute number of disability-adjusted life years (DALYs) has increased by 29% to 129 million from 1990 to 2010 [2]. Moreover, the major causes of catastrophic healthcare expenditure include IHD and its sequelae, and such burden devastates families to a point of no return to normalcy ever. From a future perspective in the USA alone, IHD-related healthcare costs were projected to rise by 41% from $126.2 billion in 2010 to $177.5 billion in 2040 [3]. The cost of long-term management of IHD is another metric that cannot be neglected. Heart failure, the most common sequela of IHD, costs an estimated $108 billion annually worldwide [4]. Similarly, for stroke there were 5·5 million (95% uncertainty interval with a range of 5·3–5·7 million) deaths and 116·4 million (111·4–121·4 million) DALYs due to stroke. There were 13·7 million (12·7–14·7 million) new stroke cases in 2016 and 80·1 million (74·1–86·3 million) prevalent cases of stroke globally in 2016 [5]. Like cardiovascular diseases (CVDs), although the age-standardized death and prevalence rates of stroke have decreased significantly over time, the overall burden of stroke has remained high and is currently second only to CVD [6]. As measured by the Barthel index, stroke patients suffer on an average at least 0.86 years with mild disability, 1.24 years with moderate disability, and 1.39 years with severe disability [7]. Such disabilities signify a severe dent to healthcare expenditure.

Despite the enormous cost in the management of cardiovascular diseases, there is also the complexity of the changing face of the endemicity in all countries. The developing countries are witnessing a vastly decreased burden of classical infectious diseases (except the COVID pandemic) and increased lifespan of individuals from 40–50 years to 70–80 years, many more experience the complications of atherosclerosis, predominantly heart attacks (the most common cause for heart failure leading to death), strokes (which debilitates the life of the affected and at least one other caretaker) and peripheral artery disease (which severely affects quality of life). Hence, if anyone escapes the early complication of death, she/he is bound to bear the burden in other forms till death.

For the last 3–4 decades, the management of CVDs was restricted to a combination of the classical drugs such as aspirin, beta-blockers, angiotensin-converting enzyme (ACE) inhibitors, and statins. However, medical interventions are now considered to modify risk factors. These interventions include: (i) lipid control without a lower limit using statins and other lipid lowering therapies such as ezetimibe and approved PCSK9 inhibitors, (ii) ACE inhibitors to control blood pressure, and (iii) therapies in patients with co-morbidities mainly type 2 diabetes and/or heart failure. Guidelines have also been established to modify risk factors, which include smoking cessation, physical activity, dietary recommendations, weight reduction and psychological and social support interventions. These secondary preventive measures modify the characteristics of a vulnerable atherosclerotic plaque leading to an increase in the incidence of erosion-induced thrombi [8]. In the USA, the secondary preventive measures have been effective in decreasing the incidence of ST-segment elevation MI (STEMI) compared to non-STEMI [9].

**Fig. 1:**
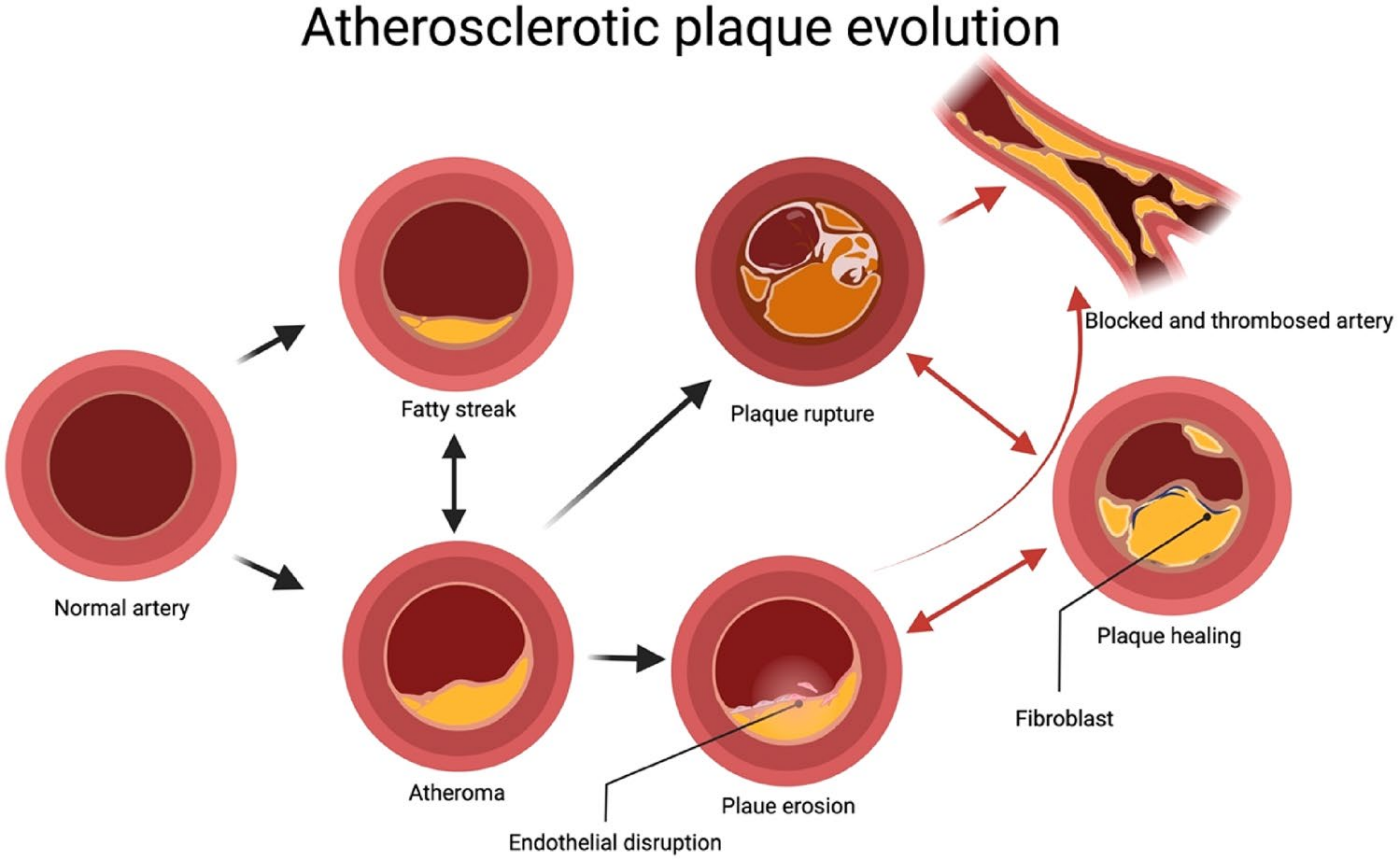
Formation of atherosclerotic plaques and their evolution. The atherosclerotic plaques progress from a fatty streak to a classic atheroma leading to either an erosion or the rupture of thin-capped fibroatheroma, which cycles between healing, thrombosis and finally to blockage of the concerned artery. Red arrows depict an association with probable clinical symptoms, while black arrows are for features of the plaques that are usually clinically silent. There could be multiple cycles of healing and rupture before an artery is blocked

Progression of CVDs is dynamic and unpredictable, which can lead to the most feared entity, MACE (major adverse cardiovascular events), that include MI, stroke, or death. Despite a guideline-based successful combination of these agents, a very high “residual risk” still exists, especially in patients with comorbidities such as polyvascular disease, diabetes, and obesity. The estimated probability of having MACE within 5 years of the onset of apparent stable angina is up to 35% [10] and that the annual repeat event risk with standard treatment may be as high as 2.5–5% [11]. In fact, the very term of “stable coronary artery disease (CAD)” is being questioned and replaced with “chronic CAD” and concepts are being targeted not to individual culprit lesions or vulnerable lesions but to the actual responsible factors, such as vulnerability of a patient [, 12, 13]. Although the occurrence of an acute coronary syndrome (ACS) typically follows the typical rupture or erosion of a plaque, a thrombosis-promoting milieu is necessary for a clinically detectable myocardial ischemia. This appears to be due to an unfortunate coalescence of prothrombotic features that include systemic inflammation, abnormal fibrinolysis, vasoconstriction, and a profound stimulus for thrombosis [14]. The strongest predictors of MACE are the overall atherosclerotic plaque burden and activity combined with all the risk factors that promote a prothrombotic milieu [15]. Hence, the focus is now largely on the “atherosclerotic disease burden” in patients with a definite "residual risk" rather than on features of individual plaques such as “vulnerability” [, 16, 17] (Fig. 1).

**Fig. 2:**
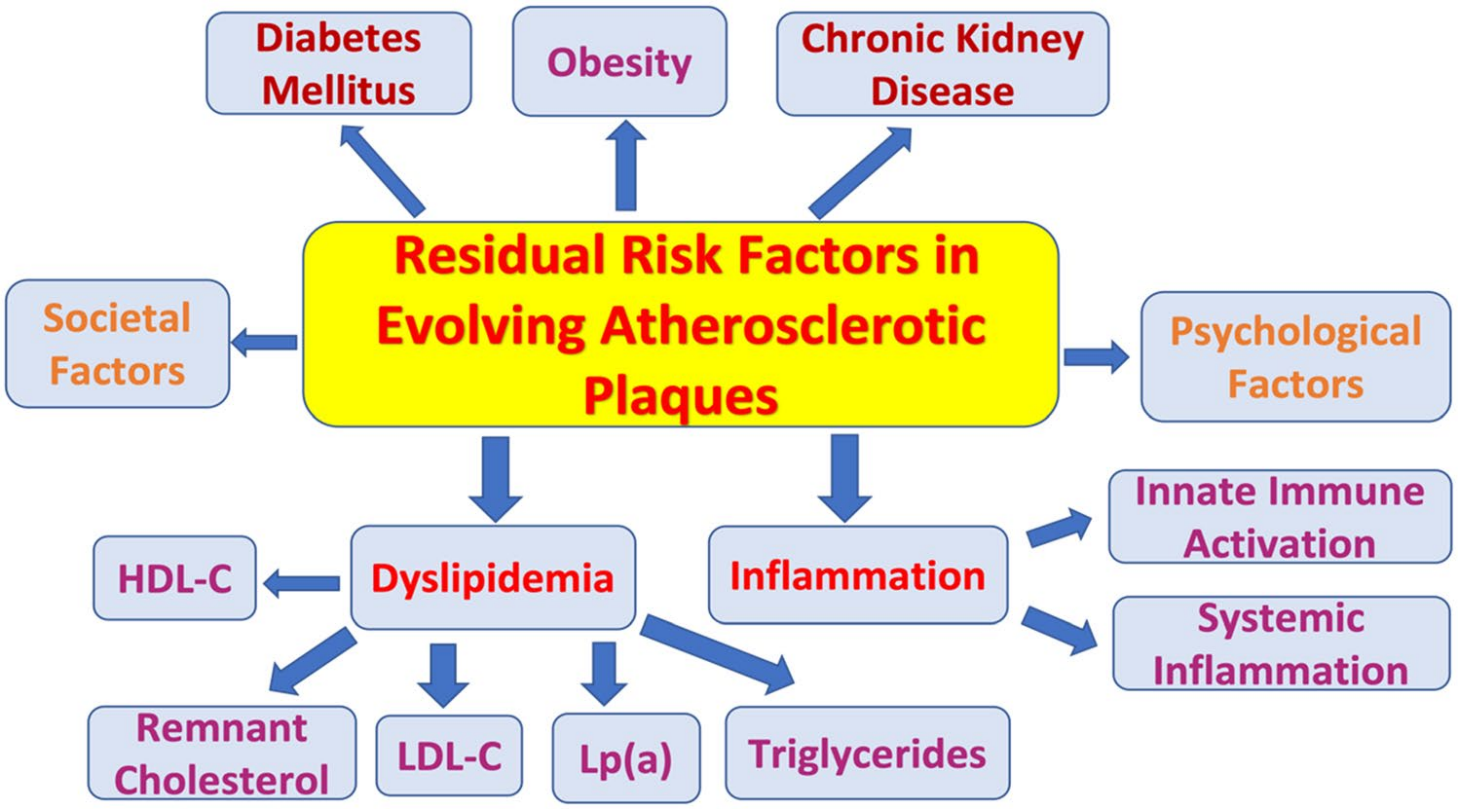
Schematic diagram showing residual risks in evolving atherosclerotic plaque

The notion of vulnerable plaque can be traced to early pathologic autopsy studies that changed the thinking of the cardiology community towards a ruptured plaque [18]. However, new contemporary data do not support the vulnerability of TCFA (thin-capped fibroatheroma; < 65 μm) to cause rupture and thrombotic events. Multiple "active" plaques are common in patients with acute coronary syndrome (ACS). In a large clinical trial (PROSPECT, Providing Regional Observations to Study Predictors of Events in the Coronary Tree), only about 5% of TCFAs as defined by intravascular ultrasound (IVUS)-based virtual histology led to acute coronary events during a 3.4-year follow-up period [19]. In this study, the lesions that were attributed to cause future MACE were only mild at baseline by angiography (mean diameter stenosis of 32 ± 21%), but not by IVUS (where the mean plaque burden was 67 ± 10% for these lesions) and progressed substantially with an angiographic mean diameter stenosis of 65 ± 16% at the time of the MACE follow-up. This means that mild lesions can progress to instability even before the information from stenotic angiograms, while > 50% angiographically stenotic lesions could stay stable for a long time. The information from such advanced techniques performed clinically has overhauled our understanding of plaque pathophysiology, when correlating to relevant clinical end points such as MACE. Even in this era of extensive lipid lowering treatments such as proprotein convertase subtilisin/kexin type 9 inhibitor (PCSKi), superior control of hypertension with ACE inhibitor (ACEi) and smoking cessation, there is a significant residual risk of MACE that exists [, 20, 21]. Here, we highlight all the residual risks contributing to the complexity of atherosclerotic disease and briefly reveal how these could contribute to the evolution of plaques that could switch from "vulnerable" to "stable" or "thrombosed" or involve multiple cycles thereof. Though the list of factors contributing to the residual risk is long (Fig. 2), in this article risks related to diabetes mellitus, obesity, chronic kidney disease, psychological and societal factors are not discussed. Rather, we critically reviewed and focused the discussion in this article on the most involved risks related to dyslipidemia and inflammation.

## Residual risks of dyslipidemias

### Low-density lipoprotein cholesterol (LDL-C)

Dyslipidemia represents a leading modifiable risk factor in the complications of atherosclerosis. An increased ratio of apolipoprotein B (apoB) to apolipoprotein A1 (apoA1) in the INTERHEART study in 52 countries with a sample size of 27,098 participants was associated with significant high mortality odds ratio compared to all other modifiable risk factors except smoking [22]. The findings from at least 4 large clinical trials [, , , 23–26] have shown significant benefits of statins. These clinical trials include: PROVE-IT (Pravastatin or Atorvastatin Evaluation and Infection Therapy; pravastatin 40 mg/day vs atorvastatin 80 mg/day) [23], TNT (Treating to New Targets; atorvastatin 10 vs atorvastatin 80 mg/day) [24], A to Z (Aggrastat-to-Zocor; placebo followed by 20 mg/day simvastatin vs 40 mg/day followed by 80 mg/day simvastatin) [25], and IDEAL (Incremental Decrease in End Points Through Aggressive Lipid-Lowering; simvastatin 20 mg titrated to 40 mg vs 80 mg/day atorvastatin) [26]. In fact, a meta-analysis of these trials has proven that intensive lipid lowering with high-dose statin therapy provides a significant benefit over standard-dose therapy for preventing predominantly nonfatal cardiovascular events [27]. The therapeutic concept of reducing CV risk by lowering LDL-C with the use of statins is proven beyond doubt and well accepted in medical practice. It has also been quantified that reducing LDL-C by 2–3 mmol/L (36–54 mg/dL) would reduce the CV risk by about 40–50% [28]. Incorporation of repeated measurements of blood pressure and cholesterol into CVD risk prediction models also improves risk prediction as garnered from a study of more than a million measurements of systolic blood pressure, total cholesterol, and high-density lipoprotein cholesterol in 191,445 adults over a median of 12 years of follow-up [29]. Despite these advances of using intensive therapy, large, randomized control trials using a combination of statin + ezetimibe or statin + PCSK9i have shown to further reduce the risk by further lowering of LDL-C without a lower limit. The common side effect of these combinations of impaired cognitive function of very low LDL-C in blood has also been shown to be not true in large, randomized studies [, , 30–32].

There is a clear shift of focus from targeted LDL-C lowering to eradicating as far as possible to achieve an optimal CV risk reduction with the tags of “even lower is even better” and “lowering LDL-C for longer duration is even better” during the rounds. Even though an aggressive LDL-C lowering approach with statins and a combination of others without a specific predefined goal (the “fire and forget” concept) is advocated clinically, the role of non-statin treatment in non-high-risk patients is completely ill-defined. Evidence of this very real residual risk even after lowering LDL-C comes from the clinical trials of IMPROVE-IT [30] and FOURIER [33] where ezetimibe and PCSK9i showed clear benefit of further reduction in LDL-C levels. It is also important to note that the cost of these additional treatments is prohibitive even in the developed countries. In contrast, novel RNA-based therapies of PCSK9 inhibition based on early clinical trial results promise up to 50–60% reduction with only two injections per year [34] pending long-term efficacy and safety data. These are also believed to improve adherence and significantly reduce the cost as the current PCSK9i approaches are financially prohibitive. However, there is a still a significant residual CV risk even with low levels of LDL-C as evidenced by meta-analyses of statin trials. In these trials, an on-treatment residual CV risk can be observed in some patients, with a staggering 5-year major event rate of 22% in patients with preexisting CVD [, 28, 34].

### High-density lipoprotein cholesterol (HDL-C)

HDL-C is an independent protective factor for CAD first seen in the Framingham Heart Study that demonstrated an inverse relation between HDL-C and the incidence of CAD [35]. There is a strong correlative evidence that higher circulating levels of HDL-C are protective in the participants of the statin clinical trials [36]. The inference of causality from observational studies where there is correlation of exposures with unequal measurement variabilities is indeed a complex issue. The association of high concentration of triglycerides (TG) with CV risk is attenuated when adjusted for HDL-C. However, this is not the case while adjusting to low HDL-C. This suggests that HDL-C was more important than TG in estimating CV risk [37]. Although HDL-C is a strong marker of risk in observational studies, it is inversely correlated to TG, which may be the true culprit.

The clinical testing of the three classes of oral agents, niacin, fibrates, and inhibitors of cholesteryl ester transfer protein (CETP) that significantly raise the levels of HDL-C in blood, in addition to lowering LDL and/or plasma TG, has been largely ineffective. These trials were either prematurely terminated [38] or showed only modest effects [39] in primary end-points. Although some clinical trials with fibrates and niacin gave variable outcomes individually, meta-analysis studies support the beneficial effects of both. However, it is difficult for post hoc analyses to clinically correlate the benefits to HDL alone. When early reports showed that transgenic over-expression of the CETP gene can induce atherosclerosis in mice [40] there was an impetus to search for molecules with CETP inhibitory activity. But concerns were expressed that the rise in HDL-C due to low CETP activity might be accompanied by a decrease in reverse cholesterol transport [41] because CETP also plays a role in the remodeling of HDL [42]. Attempts to determine whether the net effect of CETP inhibition is to promote or reduce atherogenesis have been only inconclusive till date. Despite our poor understanding of the mechanistic basis of CETP inhibitors, several promising molecules have advanced to the clinical stage, and two phase III clinical trials have reported conflicting results. The ILLUMINATE trial of torcetrapib was terminated prematurely after it became evident early in the trial that there was increased incidence of CVD events, by almost 25% (*P* = 0.001), despite increasing HDL-C by 72% and simultaneously decreasing LDL-C by 25% [43]. The Dal-OUTCOMES study of dalcetrapib (which is a less potent inhibitor than torcetrapib with no side effect on blood pressure) was stopped early due to the lack of any effect on statistical grounds [44].

The complexity of HDL role in atherosclerosis regression, based on in vitro studies, needs reconciliation with clinical evidence. When assessing the outcome of clinical trials of HDL elevators, it must be noted that, except for some of the trials, both the treatment and control groups would have included a statin. Although appropriate from the perspective of the patient well-being, this practice has inevitably made it more difficult to demonstrate efficacy in preventing CVD above and beyond the control group. There is more to mechanistically understand the complex relationship between plasma HDL-C concentration, HDL particle number, HDL subpopulation heterogeneity, molecular composition in an individual, and the ability of HDL particles to mediate cholesterol efflux from macrophages termed as, reverse cholesterol transport (RCT). Anti-atherothrombotic actions of HDL are also known to include mechanisms of antiplatelet [45], antioxidative [46], anti-inflammatory [46], anti-apoptotic [46], vasodilatory [47] and glucose metabolism [48]. These mechanisms can act at multiple stages in atherothrombosis, and hence, the role of HDL cannot be minimal. Results of the ongoing phase III clinical trials of direct HDL infusion therapies and novel CETP inhibitors such as anacetrapib and evacetrapib that can reduce LDL-C and lipoprotein (a) to 40–45% and elevate HDL-C up to 130% with or without a statin are now under investigation [, , , 49–52].

### Triglycerides and remnant cholesterol

Triglycerides and cholesterol are not soluble in plasma and hence are transported as spherical lipoprotein particles with a central core of TGs and cholesterol divided into 5 major classes based on density, which is inversely related to their size and lipid content. These are chylomicrons (CMs), very-low-density lipoprotein (VLDL), intermediate-density lipoprotein (IDL), LDL, and HDL. CMs and VLDL are the major carriers of TGs. Remnant cholesterol is the cholesterol content of triglyceride-rich lipoproteins, composed of VLDL and IDL in the fasting state and including CMs in the non-fasting state produced after lipolysis with lipoprotein lipase (LPL) and glycosylphosphatidylinositol anchored high-density lipoprotein-binding protein 1 (GPIHBP1) [53]. They essentially include all cholesterols except those in LDL and HDL. In contrast to the situation with HDL, where Mendelian randomization studies supported high remnant cholesterol rather than low HDL-C as a causal risk factor for cardiovascular disease [54], genetic polymorphisms with a statistical framework to correlate plasma lipid measures with CAD risk suggested a causal role for TG-rich lipoproteins [55].

In a post hoc analysis of the trial “Treating to New Targets,” where statins of low and high dose were compared, among the patients with LDL-C levels less than 70 mg/dL, the participants in the lowest quintile of HDL-C and high levels of TG had the highest adverse event rate (defined as first major cardiovascular event, or as death from CHD, nonfatal non-procedure-related MI, resuscitation after cardiac arrest, or fatal or nonfatal stroke) [56]. Even though as mentioned in the previous section, this residual risk was initially attributed to low HDL-C levels, the evidence for TG as the main culprit is now overwhelming [, 57, 58]. Triglyceride risk estimation has long been the most problematic due to its strong inverse relation to HDL-C and variability in its measurement, with a median variation of 23.5% compared with 4.9% for total cholesterol, 6.9% for HDL-C, and 6.5% for calculated LDL-C [59]. The inter-individual variability is mainly biological, stemming from different lifestyles, medications, metabolic abnormalities, postural effects, phlebotomy-related issues, and most importantly fasting versus non-fasting state [60]. In the large meta-analysis from “Emerging Risk Factors Collaboration (ERFC)” study, comprising 302,430 people without an initial vascular disease compiled from 68 long-term prospective studies (of Europe and North America), a total of 12,785 cases of CHD were recorded from a total of 2.79 million person-years of follow-up, showing a hazard ratio for the primary outcome (nonfatal MI and CHD death) for TG as 1.37 (95% CI, 1.31–1.42) after adjustment for non-lipid risk factors [61]. This residual risk attributed to TGs is more complex when we examined the relationship of TGs with metabolic syndrome. The risk of comorbidities such as diabetes and obesity, with an attendant insulin resistance and a high-carbohydrate diet, favors a “non-fasting” rise in triglycerides, which is correlated better than CVD risk with fasting triglycerides. In fact, hydrolysis of triglyceride rich lipoproteins (e.g., chylomicrons and VLDL) result in the generation of “atherogenic” cholesterol-enriched remnant lipoprotein particles (RLPs). Recent evidence also suggest that non-fasting triglyceride is strongly correlated with RLPs [62], and non-fasting triglyceride levels were a superior predictor of CVD risk compared to fasting levels [63]. The rise of the cluster of conditions referred to as the ‘metabolic syndrome’, which includes increased waist circumference, low HDL cholesterol, high blood pressure and raised fasting blood glucose are also typically characterized by increased plasma triglycerides. The prevalence of metabolic syndrome rose by more than 35% from 1988–1994 to 2007–2012 in the USA [64] leading to be one of the major contributors of residual risk in atherosclerosis.

### Lipoprotein (a)

Lipoprotein (a) [Lp (a)] is a unique type of low-density lipoprotein variant, which has an additional protein apo(a) only seen in humans, primates, and hedgehogs. Lp (a) has also been established as an independent risk factor almost 50 years ago [65]. Apo (a) is a homologue of plasminogen, containing a varied number of kringle 4 (KIV) domains giving rise to 34 different-sized apo (a) isoforms [66]. The concentration of Lp (a) in an individual is hugely variable, highly heritable, and is independent of environmental factors. Hence, the associated risk is attributable mainly to the genetics of the individual. Elevated levels of Lp (a) significantly associated with the recurrent ischemic events in patients who underwent percutaneous coronary intervention (PCI) [67]. Lp (a) levels are generally unaffected to traditional lipid-lowering drugs, such as the statins or fibrates except for niacin. Niacin has routinely been shown to effectively lower Lp (a) levels, when given in high doses (2–3 g/day), but these doses are associated with side effects such as headaches and liver toxicity. Apart from its role as an independent CVD biomarker, the physiological function of Lp (a) still is not completely understood [68].

Lp (a) is structurally like plasminogen and tissue plasminogen activator and hence is believed to compete with plasminogen for its binding site, leading to reduced fibrinolysis. Lp (a) can also stimulate the secretion of plasminogen activator inhibitor 1, leading to thrombogenesis [69]. Lp (a) also carries cholesterol and binds to atherogenic proinflammatory oxidized phospholipids, which can attract inflammatory cells to vessel walls leading to smooth muscle cell proliferation. In fact, the inflammation caused by Lp (a) is largely associated with the presence of oxidized phospholipids, and a specific example was demonstrated on the KIV-10 domain of apo (a) in a targeted mutagenesis experiment [70]. Oxidized phospholipids (oxPL) on Lp (a) induce the secretion of IL-8 by THP-1 macrophages [71] and addition of the monoclonal antibody E06 to bind and block the effects of oxPL on Lp (a) decreased the secretion of inflammatory cytokines, TNF-α and IL-6, by monocytes from healthy donors [72]. Based on the results of a recent clinical trial (ODYSSEY Outcomes: Evaluation of Cardiovascular Outcomes After an Acute Coronary Syndrome During Treatment with Alirocumab; NCT01663402 [73], the residual risk due to Lp (a) is convincing. In patients with recent ACS whose LDL-C is less than or around 70 mg/dL on optimized statin therapy, PCSK9 inhibition provides incremental clinical benefit only when Lp (a) concentration is at least mildly elevated [73]. Even though Lp (a) is an independent risk factor, Lp(a) and LDL-C are strongly associated in ACS patients, at least in the young (median age of 49 years), suggesting that Lp (a) might promote initiation and early development of atherosclerotic plaques which might progress aggressively in the presence of high LDL-C [74].

The appropriate management of Lp (a) is not well defined, even though there are several novel drugs and a non-pharmacological treatment in the form of Lipoprotein apheresis. The clinical benefit of Lp (a) lowering is most likely to be proportional to the absolute reduction in Lp (a) concentration. It has been estimated that large absolute reductions in Lp (a) of approximately 100 mg/dL may be required to produce benefit that is similar in magnitude to what can be achieved by lowering LDL-C level by 38 mg/dL (i.e., 1 mmol/L) [75]. All the pharmacological agents, including PCSK inhibitors, CETP inhibitors, Niacin and Mipomersen, can reduce Lp (a) to utmost 30% except ASOs (antisense oligonucleotides), which can reduce Lp (a) absolute levels up to 90% [76]. The ongoing study “Lp (a)HORIZON” will hopefully provide a clear answer in 2024 as to whether selective Lp (a) lowering with ASO reduces the risk of major CV events (NCT04023552) [77].

## Residual inflammatory risk

Addressing the problem of residual risk after statin therapy has proven complex as differing biological processes drive recurrent events in different patients. On the one hand, individuals treated with statins who have persistently elevated levels of atherogenic lipoproteins represent a specific group with residual cholesterol risk where additional lipid-lowering therapies, such as ezetimibe and PCSK9i, are likely to be effective. On the other hand, most patients treated with a higher intensity statin therapy will achieve 50%–85% reductions in LDL-C and apolipoprotein B. For these individuals in whom cholesterol is no longer the primary problem, the translational research community has long been concerned about the role of innate and acquired immune function in driving recurrent atherosclerotic events. Such patients have recently been described as having residual inflammatory risk and might benefit from anti-inflammatory treatments rather than further lipid lowering. It is only in the last few decades that evidence from experimental and clinical studies has lent support to the inflammatory hypothesis, in the pathogenesis of atherosclerotic disease in conjunction with or beyond elevated lipid levels. The recent results of CANTOS trial (Canakinumab Anti-Inflammatory Thrombosis Outcome Study) have now provided an unequivocal support for the inflammatory hypothesis of atherosclerosis where clinical evidence that controlling vascular inflammation independently of lipid lowering could lower the rates of recurrent cardiovascular events in a large phase 3 trial [78]. Inflammatory signals probably cause plaque instability, which could result in plaque rupture, fissuring, or erosion leading to a thrombotic response that causes MI. Yet, pure anti-inflammatory drugs have never been used to treat patients with CAD.

Elevated leukocyte count has traditionally been correlated with cardiovascular disease since the 1920s [79]. Although the association between leukocytosis and increased morbidity and mortality of ischemic vascular disease is robust [, 80, 81], it is not clear whether the association is causal in nature. Sterile inflammation that ensues immediately after an acute coronary event has been consistently shown to cause serious adverse effects whose pathogenesis has been well researched [, 82, 83]. The myeloid compartment of the WBCs, namely monocytes [84], neutrophils [, 85, 86] and platelets [87], is the predominant players that influence CAD initiation or progression and impair the regression of atheromatous plaques. In this context, it is not surprising to note that aspirin by virtue of its inhibition to platelet aggregation by blocking thromboxane A_2_ formation has been used for several decades as the gold-standard in secondary prevention of CAD [88].

## The evolving pathogenesis of atherosclerosis contributing to the residual risk

Atherosclerosis represents a major component of IHD and stroke that usually develop in branches and curved spaces of medium-sized arteries where there is a turbulent flow of blood [89]. It is a multifactorial disease, which involves chronic inflammation [, 90, 91], lipid metabolism and accumulation that was identified as early as the 1950s [92], oxidative stress [93], genetic predisposition [94], immune disorders [95], epigenetics [96], and multiple non-genetic risk factors such as environmental pollution, occupational exposure, smoking, mental health, diet, and lifestyle [97].

The prevailing theory of the initiation of atherosclerosis is a “multiple hit” hypothesis, which considers all these insults acting together. After initial endothelial injury due to persistent turbulent flow of variable viscosity of blood, endothelial dysfunction in the form of altered expression of adhesion molecules is the first step in atherosclerosis. This results in increased adhesion/transmigration of monocytes, which then differentiate into macrophages that can take up lipids to form a characteristic cell type called a “foam cell,” leading to a fatty streak on the internal surface of the arteries. Subsequently, vascular smooth muscle cells (VSMCs) residing in the media layer migrate to sub-endothelial space, proliferate, and form an aberrant extracellular matrix leading to fibroatheroma formation and atherosclerotic plaque. After a significant accumulation of foam cells and aberrant VSMCs and as the plaque grows internally, a large necrotic core forms that can be covered by a layer of fibrous tissue forming a fibrous cap. After years of accumulation of these factors, changes in the thickness of the caps ensue leading to a vulnerable plaque, which when ruptures can cause the thrombus to block the blood flow or dislodge and travel to a distal narrower vessel leading to ischemic stroke or MI [98].

The classic concept of a “vulnerable plaque” has garnered the attention for the last few decades and is mainly dependent on the decreased synthesis and increased breakdown of interstitial collagen in the fibrous cap of the plaque [99]. The smooth muscle cells are the major source of extracellular matrix proteins and foam cells in the arteries. Numerous inflammatory cytokines, such as interferon (IFN)-*γ*, tumor necrosis factor (TNF)-*α*,*β* and others, can and do inhibit the production of interstitial collagen, while macrophages and other inflammatory T cells cause the expression of various matrix metalloproteinases (MMPs) that can thin the caps of TCFAs [, 100, 101]. Most importantly, the potent procoagulant molecule "tissue factor" is released from the plaque triggering thrombotic complications. Systemic inflammatory status also contributes to excess fibrin production, which is the precursor of clots and reduction of clot inhibiting factors such as plasminogen activator inhibitor-1 (PAI-1). This chain of pathogenic events gained acceptance for quite a few decades heightening the prominence of the role of TCFAs in ACS. Nevertheless, as highlighted in the previous sections a significant residual burden of ACS events persists even after measures are taken to fight plaque vulnerability. It has also been the experience since the last two decades that only less than 20% of TCFAs cause ACS fatalities [102]. The recent results of PROSPECT trial substantiate the same and reduced that to 5%. There are several reasons that can be said of why TCFAs do not actually cause most MACEs. For example, it is well known that TCFAs are seldom seen in solitary but are multiple in a coronary bed and are also wildly present in other vascular beds in the same individual, persisting for years without causing a clinical event. The changing demographic of the population with increase in the number of revascularizations, availability to better versions of lipid lowering drugs, evidence of decreasing incidence of STEMI vs non-STEMI in the developed world all point to an attendant change in the modification of the TCFAs into stable atherosclerotic plaques [, 16, 103].

Cerebrovascular atherosclerotic plaques have been known to be become more stable plaques compared to what they were a decade ago, though this may well be due to high activity of antioxidant enzymes in intracranial arteries [104]. More recently, questions about plaque biology are being more rigorously addressed by intravascular ultrasound (IVUS), which correlates radiofrequency spectral analysis with plaque characteristics leading to what is termed as "virtual histology" in a live patient [105]. Imaging of the arteries with IVUS and virtual histology have proven to be far more useful than conventional angiography [, 106, 107]. Other advanced techniques being applied with greater confidence are optical coherence tomography (OCT) and near-infrared spectroscopy. Grayscale and integrated backscatter IVUS imaging are some offshoots that have shown promise in allowing the live quantification of sensitive histological parameters such as total lipid volume and fibrous cap thickness [108]. These novel techniques have become surrogate end points of current major clinical trials and are most likely to inform on important characteristics of plaque evolution that are directly being measured, such as plaque size, plaque composition, and arterial remodeling. An interesting mechanistic concept of a longitudinal necrotic shafts near a TCFA rupture led to excavation of the thrombogenic material into the lumen, resulting in thrombosis and acute vessel closure was proposed [109].

The re-evaluation of the vulnerable plaque concept has ignited interest in a different pathological entity, namely “plaque erosion,” which so far has been relegated to be as not so important in ACS. As early as 1996, plaque erosion of “proteoglycan-rich and smooth muscle cell-rich” plaques, which lack a lipid core and are not prone to plaque rupture, has been frequently found in as many as 44% of sudden death cases in ACS [110]. Superficial plaque erosion can correctly be detected in up to 40% of patients with CAD undergoing percutaneous interventions [111]. This was made possible due to the growing use of OCT and IVUS techniques during coronary angiography, which have resulted in a more precise and improved definition of atherosclerotic lesion morphology, and a better identification of lesions with a thin-cap and a large lipidic/necrotic core. The presence of thick fibrous caps with small necrotic cores at sites of angiographically non-obstructive lesions can be dangerously misleading. This is because lesions with supposed superficial “erosion” can potentially masquerade as “stable” plaques before the occurrence of thrombosis. It is believed that the luminal thrombus of eroded plaques is often overlaid without any endothelial cells and is also rich in proteoglycans and smooth muscle cells, unlike vulnerable plaques where the presence of macrophages and lymphocytes is predominant. Unlike “vulnerable” plaques, “eroded” plaques often lack a necrotic core or, and if the latter is present, it is usually buried deep under a thick fibrous cap, which makes it invisible to in vivo percutaneous interventional techniques. Such detailed characterization of culprit lesions is now strengthening the concept that the superficial erosion might associate more commonly with non-STEMI and is also supported by contemporary studies [, 112, 113].

Is lipid lowering due to the extensive use of statins responsible for the change in the outcomes of many TCFAs and “vulnerable plaques”? Following the occurrence of a myocardial event, subsequent lipid lowering only reduces luminal stenoses assessed by angiography by an average of at most to only a few percent. However, it is well documented by numerous clinical trials that the fall in MACE produced by statins dwarfs these small changes in angiographically measured stenoses by many orders of magnitude. This apparent paradox has shifted the current concepts in pathogenesis from vulnerable plaques to plaque erosion and repeated cycles of stabilization and destabilization, which is also termed as "plaque evolution." These concepts are very well supported by numerous animal studies in terms of mechanisms, where an increase in collagen content was confirmed with in situ histomorphology observations made in primates [114]. Markers of inflammation, significantly subside after lipid lowering, in parallel with a reduction in the expression of interstitial collagenases implicated in collagen breakdown and the linking of inflammation with weakening of the fibrous cap in the plaque [, , , , , 115–120]. Further studies show a decrease in tissue factor expression, establishing a likely mechanism of reduced thrombogenicity of plaques following lipid lowering and decrease atherothrombotic complications after superficial plaque erosion by reducing serum oxysterols [121]. Studies in Watanabe heritable hyperlipidemic rabbits treated with HMG-CoA reductase inhibitors for lipid lowering reported altered plaque biology by reducing proliferation and activation of macrophages [122]. However, how does one explain the “residual risks” of atherosclerotic events after stabilization of plaques due to statins? Contemporary data suggest that plaque erosion which used to contribute to one fifth of ACS events now appears to account for more than one-third of ACS events [112]. In contrast to ruptured plaques, eroded plaques are rich in proteoglycans, glycosaminoglycans have little to no lipid core, increased products of innate immune cells such as neutrophil extracellular traps, many smooth muscle cells and platelet-rich thrombi rather than fibrin-rich red thrombi typically associated with TCFAs [123]. However, the most important aspect of plaque erosion could be the loss of endothelial integrity, which could amplify and propagate thrombi in atheroma of intact fibrous caps. There are many characteristic features that are different between a plaque prone to plaque rupture vs. an eroded plaque, which are listed in Table 1.

**Table 1:** Contrasting characteristics of plaque rupture vs plaque erosion

Plaque rupture	Plaque erosion
A central core of lipids and inflammatory cells	No central core
Lipid rich	Lipid poor
Thin cap above the core and at corners	Thick fibrous tissue with endothelial erosion
Endothelial discontinuity due to pressure	Endothelial discontinuity due to activation and innate immune response
Interstitial collagen breakdown	Proteoglycan, glycosaminoglycan and hyaluronan rich
Apoptosis of smooth muscle cells	Apoptosis of endothelial cells
Abundance of inflammatory cells typically macrophages	Paucity of inflammatory cells but usually neutrophils
Typically show red thrombus (Fibrin rich)	Typically have white thrombus (platelet rich)
High LDL	High triglycerides
TLR-4 signaling	TLR-2 signaling
Activity of MMP-1, MMP-8 and MMP-13	Activity of MMP-2
Typical incidence in males and elderly	Typical incidence in females and young patients

Early hypotheses proposed mainly two mechanisms that could heighten the risk of endothelial desquamation in the context of superficial erosion: scission of the tethers of the basal surface of the endothelial cell to the subjacent basement membrane and endothelial cell death [8]. Collagen IV is the major constituent of the basement membrane, and hence, interstitial collagenases that attack collagens I and III likely contribute to plaque rupture, while collagenases that target collagen IV could be responsible for plaque erosion. MMP-2 (gelatinase A), the major type IV collagenase, rather than MMP-1, MMP-8, and MMP-13 MMP-1, MMP-8 and MMP-13 that have been shown to be important in plaque rupture that attack collagens I and III, could participate in plaque erosion [, 124, 125]. Various inflammatory cytokines like TNF-*α*, IL-1*α*, or IL-1*β* and Ox-LDL can increase the levels of MT1-MMP promoting matrix degradation by activating pro-MMP-2 [126]. Precursor MMP-2 potentiates activation of pro-MMP-13 by MT1-MMP, and active MMP-13 can in turn activate MMP-2 and MMP-gelatinase zymogens, thereby indicating an activation cascade of three members of the MMP family [127]. Thus, increased expression of MT1-MMP can favor digestion of native interstitial collagens by MMP-13, as well as continued degradation of partially degraded collagens due to gelatinase activity of MMP-2, and proteolysis of basement membrane type IV collagen by MMP-2.

As to the second mechanism of endothelial cell death, hypochlorous acid an important oxidant and the product of neutrophil myeloperoxidase (MPO) can promote apoptosis of endothelial cells along with a huge amount of tissue factor gene expression as shown by human endothelial cells in vitro [128]. Studies as early as year 2000 of endarterectomy specimens showed that luminal endothelial cell apoptosis may be a major determinant of plaque erosion and thrombosis [129]. Early studies also established that increased endothelial expression of TLR2 at sites of disturbed blood flow may exacerbate early atherogenic events [130]. An elegant study recently showed that flow disturbance, neutrophils, and TLR2 signaling contribute to superficial erosion in mice that is garnering attention in the statin era [131]. The quest for TLR2 ligands of endogenous origin has implicated hyaluronan fragments, their receptor CD44 and proteoglycans, where it is known that these accumulate at sites of superficial erosion [132]. It has been hypothesized that the loss of endothelium and exposure to a potentially procoagulant versican–hyaluronan matrix may be largely responsible for plaque erosion [133]. These two mechanisms of desquamation of endothelial cells and endothelial apoptosis have been used to postulate a two-hit thesis, where activation and desquamation of endothelial cells are caused by low-level innate immune activation along with flow disturbance, and this is followed by chemokine secretion and recruitment of more immune cells. These could aggravate injury by neutrophil extracellular traps, which are deposited at the sites of erosion, a well-known phenomenon termed as NETosis. Interestingly, lipids can trigger or facilitate the membrane-related changes that result in endothelial cell death [134]. An in vitro study confirmed this sequence of events in superficial erosion where TLR2 stimulation followed by neutrophil engagement rendered smooth muscle cell-rich plaques susceptible to superficial erosion and thrombotic complications by inducing ER stress, apoptosis, and favoring endothelial desquamation [135]. It is not surprising that the approaches of strong anti-platelet therapy can combat the erosion-associated platelet-rich white thrombi than those associated with rupture of TCFAs, for which the therapy was brought in vogue [136].

The other not so well studied aspect of the evolution of atherosclerotic plaques is “plaque healing.” It is well known that most episodes of plaque rupture or thrombotic events associated with plaque erosion are clinically silent [137]. These disruptions are usually followed by a healing process that prevents significant intraluminal thrombosis. Plaque disruption triggers a repair response with proliferation of smooth muscle cells, which migrate from the tunica media to the intima. A two-year follow-up study of the evolution of spontaneous coronary atheromatous plaque ruptures that are clinically significant without significant stenosis detected on first ACS had healed without significant plaque modification in 50% of cases with medical therapy [138]. The introduction of new imaging methods such as IVUS-VH, OCT, and cardiovascular magnetic resonance imaging has enabled the detection and assessment of plaque healing in vivo [, 139, 140]. Based on these studies, the prevalence of healed plaques is found to be considerably higher among patients with chronic manifestations than those in patients with ACS events [, 139, 141]. This is supported by histological studies on non-culprit lesions in patients who died of MI, where more than half of them had healed coronary plaques [137]. The prevalence and occurrence of healed atherosclerotic plaques were also studied by OCT imaging at culprit sites of patients with ACS, where the healed plaques were defined as “plaques with one or more layers of differing optical density with a clear demarcation of underlying tissues and background” as assessed by at least two independent reviewers [142]. The diagnostic accuracy of OCT-based identification of healed plaques has already been evaluated with histopathological comparison in an autopsy-based study [143]. Healed plaques have also been detected using IVUS-VH where out of a small sample of 20 IVUS-VH defined thin-cap fibroatheromas 75% of which were categorized to have been healed [144]. A comprehensive study using serial imaging with both IVUS-VH and OCT showed that lesion progression can be categorized to distinct OCT morphologies that correlate to changes in plaque mass or vessel remodeling [145]. In fact, the high resolution of OCT allows the distinction between plaque rupture or erosion, thin cap fibroatheromas, and surprisingly even macrophage infiltration in most of the layered plaques. Bright spots in IVUS and OCT images have interestingly been correlated with a variety of plaque components like macrophages, which cause sharp changes in the index of refraction [146]. The healing of plaques is a natural outcome of statin therapy [147], or other lipid-lowering therapies [148] in atherosclerotic plaques of coronary or carotid arteries. Intensive anti-platelet therapy was effective in stabilizing plaque erosion, with a sustained reduction in thrombosis at one month and complete healing at one year [136]. Vergallo et al. [149] have recently summarized the various aspects influencing the healing process and elaborate on mechanisms and therapeutic implications of plaque healing. Additionally, there is potential role of non-coding RNAs in the regulation of plaque progression and remodeling of extracellular matrix in atherosclerotic plaque [150].

## Conclusion

In summary, there is a definite residual risk of atherosclerosis in cardiovascular and cerebrovascular diseases even after significant advances in therapeutics, leading to a shift in the endemicity patterns of these diseases in both developed and developing countries. The major contributors of this residual risk can still be attributed to the different forms of lipids causing the ever-changing features of atherosclerotic plaques, and certain acute inflammatory milieu that contribute to sudden changes in plaque biology. These affect the plaque biology and modulate the underlying pathology that constantly leads to what is termed as “evolving atherosclerotic plaques.” It can be said that once atherosclerotic event starts in an artery, it never stays static but keeps changing as with everything else in life. The classic picture of a fatty streak progressing into an atheroma, then leading to either a stable or vulnerable plaque, is no longer an accepted theme in contemporary times. The atherosclerotic plaque is constantly changing and evolving; hence, alternate concepts like plaque erosion, “longitudinal necrotic shafts,” or an impaired healing process with "layered plaques" may be necessary that could lead us to appreciate the complexity and an improved understanding of atherosclerotic plaques that result in residual risks. The introduction of novel live imaging methods such as IVUS-VH, OCT and cardiovascular magnetic resonance imaging, which are also being used as surrogate end points in clinical trials, could lead to a better understanding of the relationship between residual risks and evolving plaques.

## Data Availability

Not applicable since the information is gathered from published articles.

## References

[1] Dai H, Much AA, Maor E, Asher E, Younis A, Xu Y (2020). Global, regional, and national burden of ischemic heart disease and its attributable risk factors, 1990–2017: results from the global burden of disease study 2017. Eur Heart J Qual Care Clin Outcomes.

[2] Moran AE, Tzong KY, Forouzanfar MH, Rothy GA, Mensah GA, Ezzati M (2014). Variations in ischemic heart disease burden by age, country, and income: the global burden of diseases, injuries, and risk factors 2010 study. Glob Heart.

[3] Odden MC, Coxson PG, Moran A, Lightwood JM, Goldman L, Bibbins-Domingo K (2011). The impact of the aging population on coronary heart disease in the United States. Am J Med.

[4] Cook C, Cole G, Asaria P, Jabbour R, Francis DP (2014). The annual global economic burden of heart failure. Int J Cardiol.

[5] Collaborators GBDS (2019). Global, regional, and national burden of stroke, 1990–2016: a systematic analysis for the Global Burden of Disease Study 2016. Lancet Neurol.

[6] Group GBDNDC (2017). Global, regional, and national burden of neurological disorders during 1990–2015: a systematic analysis for the global burden of disease study 2015. Lancet Neurol.

[7] Hung MC, Hsieh CL, Hwang JS, Jeng JS, Wang JD (2013). Estimation of the long-term care needs of stroke patients by integrating functional disability and survival. PLoS ONE.

[8] Libby P, Pasterkamp G, Crea F, Jang IK (2019). Reassessing the mechanisms of acute coronary syndromes. Circ Res.

[9] Yeh RW, Sidney S, Chandra M, Sorel M, Selby JV, Go AS (2010). Population trends in the incidence and outcomes of acute myocardial infarction. N Engl J Med.

[10] Clayton TC, Lubsen J, Pocock SJ, Voko Z, Kirwan BA, Fox KA (2005). Risk score for predicting death, myocardial infarction, and stroke in patients with stable angina, based on a large randomised trial cohort of patients. BMJ.

[11] Hoogeveen RM, Hanssen NMJ, Brouwer JR, Mosterd A, Tack CJ, Kroon AA (2022). The challenge of choosing in cardiovascular risk management. Neth Heart J.

[12] Fox KAA, Metra M, Morais J, Atar D (2020). The myth of 'stable' coronary artery disease. Nat Rev Cardiol.

[13] Naghavi M, Libby P, Falk E, Casscells SW, Litovsky S, Rumberger J (2003). From vulnerable plaque to vulnerable patient: a call for new definitions and risk assessment strategies: Part II. Circulation.

[14] Arbab-Zadeh A, Nakano M, Virmani R, Fuster V (2012). Acute coronary events. Circulation.

[15] Greenland P, Knoll MD, Stamler J, Neaton JD, Dyer AR, Garside DB (2003). Major risk factors as antecedents of fatal and nonfatal coronary heart disease events. JAMA.

[16] Mitra AK, Dhume A, Agrawal DK (2004). Vulnerable plaques: Ticking of the time bomb. Can J Physiol Pharmacol.

[17] Arbab-Zadeh A, Fuster V (2015). The myth of the "vulnerable plaque": transitioning from a focus on individual lesions to atherosclerotic disease burden for coronary artery disease risk assessment. J Am Coll Cardiol.

[18] Davies MJ (1996). Stability and instability: two faces of coronary atherosclerosis: the Paul Dudley White lecture 1995. Circulation.

[19] Stone GW, Maehara A, Lansky AJ, de Bruyne B, Cristea E, Mintz GS (2011). A prospective natural-history study of coronary atherosclerosis. N Engl J Med.

[20] Bohula EA, Giugliano RP, Leiter LA, Verma S, Park JG, Sever PS (2018). Inflammatory and cholesterol risk in the fourier Trial. Circulation.

[21] Pradhan AD, Aday AW, Rose LM, Ridker PM (2018). Residual inflammatory risk on treatment with PCSK9 inhibition and statin therapy. Circulation.

[22] Yusuf S, Hawken S, Ounpuu S, Dans T, Avezum A, Lanas F (2004). Effect of potentially modifiable risk factors associated with myocardial infarction in 52 countries (the interheart study): case-control study. Lancet.

[23] Cannon CP, Braunwald E, McCabe CH, Rader DJ, Rouleau JL, Belder R (2004). Intensive versus moderate lipid lowering with statins after acute coronary syndromes. N Engl J Med.

[24] Deedwania P, Barter P, Carmena R, Fruchart JC, Grundy SM, Haffner S (2006). Reduction of low-density lipoprotein cholesterol in patients with coronary heart disease and metabolic syndrome: analysis of the Treating to New Targets study. Lancet.

[25] Murphy SA, Cannon CP, Wiviott SD, de Lemos JA, Blazing MA, McCabe CH (2007). Effect of intensive lipid-lowering therapy on mortality after acute coronary syndrome (a patient-level analysis of the aggrastat to zocor and pravastatin or atorvastatin evaluation and infection therapy-thrombolysis in myocardial infarction 22 trials). Am J Cardiol.

[26] Pedersen TR, Faergeman O, Kastelein JJ, Olsson AG, Tikkanen MJ, Holme I (2004). Design and baseline characteristics of the incremental decrease in end points through aggressive lipid lowering study. Am J Cardiol.

[27] Cannon CP, Steinberg BA, Murphy SA, Mega JL, Braunwald E (2006). Meta-analysis of cardiovascular outcomes trials comparing intensive versus moderate statin therapy. J Am Coll Cardiol.

[28] Cholesterol Treatment Trialists C, Baigent C, Blackwell L, Emberson J, Holland LE, Reith C (2010). Efficacy and safety of more intensive lowering of LDL cholesterol: a meta-analysis of data from 170,000 participants in 26 randomised trials. Lancet.

[29] Paige E, Barrett J, Pennells L, Sweeting M, Willeit P, Di Angelantonio E (2017). Use of repeated blood pressure and cholesterol measurements to improve cardiovascular disease risk prediction: an individual-participant-data meta-analysis. Am J Epidemiol.

[30] Cannon CP, Blazing MA, Giugliano RP, McCagg A, White JA, Theroux P (2015). Ezetimibe added to statin therapy after acute coronary syndromes. N Engl J Med.

[31] Koren MJ, Sabatine MS, Giugliano RP, Langslet G, Wiviott SD, Kassahun H (2017). Long-term low-density lipoprotein cholesterol-lowering efficacy, persistence, and safety of evolocumab in treatment of hypercholesterolemia: results up to 4 years from the open-label OSLER-1 extension study. JAMA Cardiol.

[32] Giugliano RP, Mach F, Zavitz K, Kurtz C, Im K, Kanevsky E (2017). Cognitive function in a randomized trial of evolocumab. N Engl J Med.

[33] Sabatine MS, Giugliano RP, Keech AC, Honarpour N, Wiviott SD, Murphy SA (2017). Evolocumab and clinical outcomes in patients with cardiovascular disease. N Engl J Med.

[34] Ray KK, Wright RS, Kallend D, Koenig W, Leiter LA, Raal FJ (2020). Two phase 3 trials of inclisiran in patients with elevated LDL cholesterol. N Engl J Med.

[35] Castelli WP (1988). Cholesterol and lipids in the risk of coronary artery disease–the Framingham heart study. Can J Cardiol.

[36] Jafri H, Alsheikh-Ali AA, Karas RH (2010). Meta-analysis: statin therapy does not alter the association between low levels of high-density lipoprotein cholesterol and increased cardiovascular risk. Ann Intern Med.

[37] Phillips AN, Smith GD (1991). How independent are “independent” effects? Relative risk estimation when correlated exposures are measured imprecisely. J Clin Epidemiol.

[38] Group HTC (2013). HPS2-THRIVE randomized placebo-controlled trial in 25 673 high-risk patients of ER niacin/laropiprant: trial design, pre-specified muscle and liver outcomes, and reasons for stopping study treatment. Eur Heart J.

[39] Jun M, Foote C, Lv J, Neal B, Patel A, Nicholls SJ (2010). Effects of fibrates on cardiovascular outcomes: a systematic review and meta-analysis. Lancet.

[40] Marotti KR, Castle CK, Boyle TP, Lin AH, Murray RW, Melchior GW (1993). Severe atherosclerosis in transgenic mice expressing simian cholesteryl ester transfer protein. Nature.

[41] Fielding CJ, Havel RJ (1996). Cholesteryl ester transfer protein: friend or foe?. J Clin Invest.

[42] Hennessy LK, Kunitake ST, Kane JP (1993). Apolipoprotein A-I-containing lipoproteins, with or without apolipoprotein A-II, as progenitors of pre-beta high-density lipoprotein particles. Biochemistry.

[43] Barter PJ, Caulfield M, Eriksson M, Grundy SM, Kastelein JJ, Komajda M (2007). Effects of torcetrapib in patients at high risk for coronary events. N Engl J Med.

[44] Schwartz GG, Olsson AG, Abt M, Ballantyne CM, Barter PJ, Brumm J (2012). Effects of dalcetrapib in patients with a recent acute coronary syndrome. N Engl J Med.

[45] Calkin AC, Drew BG, Ono A, Duffy SJ, Gordon MV, Schoenwaelder SM (2009). Reconstituted high-density lipoprotein attenuates platelet function in individuals with type 2 diabetes mellitus by promoting cholesterol efflux. Circulation.

[46] Kontush A, Therond P, Zerrad A, Couturier M, Negre-Salvayre A, de Souza JA (2007). Preferential sphingosine-1-phosphate enrichment and sphingomyelin depletion are key features of small dense HDL3 particles: relevance to antiapoptotic and antioxidative activities. Arterioscler Thromb Vasc Biol.

[47] Besler C, Heinrich K, Rohrer L, Doerries C, Riwanto M, Shih DM (2011). Mechanisms underlying adverse effects of HDL on eNOS-activating pathways in patients with coronary artery disease. J Clin Invest.

[48] Drew BG, Rye KA, Duffy SJ, Barter P, Kingwell BA (2012). The emerging role of HDL in glucose metabolism. Nat Rev Endocrinol.

[49] Tardif JC, Ballantyne CM, Barter P, Dasseux JL, Fayad ZA, Guertin MC (2014). Effects of the high-density lipoprotein mimetic agent CER-001 on coronary atherosclerosis in patients with acute coronary syndromes: a randomized trial. Eur Heart J.

[50] Tricoci P, D'Andrea DM, Gurbel PA, Yao Z, Cuchel M, Winston B (2015). Infusion of reconstituted high-density lipoprotein, CSL112, in patients with atherosclerosis: safety and pharmacokinetic results from a phase 2a randomized clinical trial. J Am Heart Assoc.

[51] Bowman L, Hopewell JC, Chen F, Wallendszus K, Stevens W, Group HTRC (2017). Effects of anacetrapib in patients with atherosclerotic vascular disease. N Engl J Med.

[52] Nurmohamed NS, Ditmarsch M, Kastelein JJP (2021). CETP-inhibitors: from HDL-C to LDL-C lowering agents?. Cardiovasc Res.

[53] Beigneux AP, Davies BS, Gin P, Weinstein MM, Farber E, Qiao X (2007). Glycosylphosphatidylinositol-anchored high-density lipoprotein-binding protein 1 plays a critical role in the lipolytic processing of chylomicrons. Cell Metab.

[54] Varbo A, Benn M, Tybjaerg-Hansen A, Jorgensen AB, Frikke-Schmidt R, Nordestgaard BG (2013). Remnant cholesterol as a causal risk factor for ischemic heart disease. J Am Coll Cardiol.

[55] Do R, Willer CJ, Schmidt EM, Sengupta S, Gao C, Peloso GM (2013). Common variants associated with plasma triglycerides and risk for coronary artery disease. Nat Genet.

[56] Barter P, Gotto AM, LaRosa JC, Maroni J, Szarek M, Grundy SM (2007). HDL cholesterol, very low levels of LDL cholesterol, and cardiovascular events. N Engl J Med.

[57] Nordestgaard BG, Varbo A (2014). Triglycerides and cardiovascular disease. Lancet.

[58] Triglyceride Coronary Disease Genetics Consortium and Emerging Risk Factors Collaboration (2010). Triglyceride-mediated pathways and coronary disease: collaborative analysis of 101 studies. Lancet.

[59] Bachorik PS, Ross JW (1995). National cholesterol education program recommendations for measurement of low-density lipoprotein cholesterol: executive summary the national cholesterol education program working group on lipoprotein measurement. Clin Chem.

[60] Miller M, Stone NJ, Ballantyne C, Bittner V, Criqui MH, Ginsberg HN (2011). Triglycerides and cardiovascular disease: a scientific statement from the American Heart Association. Circulation.

[61] Emerging Risk Factors C, Di Angelantonio E, Sarwar N, Perry P, Kaptoge S, Ray KK (2009). Major lipids, apolipoproteins, and risk of vascular disease. JAMA.

[62] Nordestgaard BG, Benn M, Schnohr P, Tybjaerg-Hansen A (2007). Nonfasting triglycerides and risk of myocardial infarction, ischemic heart disease, and death in men and women. JAMA.

[63] Bansal S, Buring JE, Rifai N, Mora S, Sacks FM, Ridker PM (2007). Fasting compared with nonfasting triglycerides and risk of cardiovascular events in women. JAMA.

[64] Moore JX, Chaudhary N, Akinyemiju T (2017). Metabolic syndrome prevalence by race/ethnicity and sex in the United States, national health and nutrition examination survey, 1988–2012. Prev Chronic Dis.

[65] Berg K (1963). A new serum type system in man–the Lp system. Acta Pathol Microbiol Scand.

[66] Gaw A, Hobbs HH (1994). Molecular genetics of lipoprotein (a): new pieces to the puzzle. Curr Opin Lipidol.

[67] Yoon YH, Ahn JM, Kang DY, Lee PH, Kang SJ, Park DW (2021). Association of lipoprotein(a) with recurrent ischemic events Following percutaneous coronary intervention. JACC Cardiovasc Interv.

[68] Nordestgaard BG, Chapman MJ, Ray K, Boren J, Andreotti F, Watts GF (2010). Lipoprotein (a) as a cardiovascular risk factor: current status. Eur Heart J.

[69] Banach M, Aronow WS, Serban MC, Rysz J, Voroneanu L, Covic A (2015). Lipids, blood pressure and kidney update 2015. Lipids Health Dis.

[70] Leibundgut G, Scipione C, Yin H, Schneider M, Boffa MB, Green S (2013). Determinants of binding of oxidized phospholipids on apolipoprotein (a) and lipoprotein (a). J Lipid Res.

[71] Scipione CA, Sayegh SE, Romagnuolo R, Tsimikas S, Marcovina SM, Boffa MB (2015). Mechanistic insights into Lp(a)-induced IL-8 expression: a role for oxidized phospholipid modification of apo(a). J Lipid Res.

[72] van der Valk FM, Bekkering S, Kroon J, Yeang C, Van den Bossche J, van Buul JD (2016). Oxidized phospholipids on lipoprotein(a) elicit arterial wall inflammation and an inflammatory monocyte response in humans. Circulation.

[73] Schwartz GG, Szarek M, Bittner VA, Diaz R, Goodman SG, Jukema JW (2021). Lipoprotein (a) and benefit of PCSK9 inhibition in patients with nominally controlled LDL cholesterol. J Am Coll Cardiol.

[74] Afshar M, Pilote L, Dufresne L, Engert JC, Thanassoulis G (2016). Lipoprotein (a) interactions with low-density lipoprotein cholesterol and other cardiovascular risk factors in premature acute coronary syndrome (ACS). J Am Heart Assoc.

[75] Burgess S, Ference BA, Staley JR, Freitag DF, Mason AM, Nielsen SF (2018). Association of LPA Variants with risk of coronary disease and the implications for lipoprotein(a)-lowering therapies: a mendelian randomization analysis. JAMA Cardiol.

[76] Rhainds D, Brodeur MR, Tardif JC (2021). Lipoprotein (a): when to measure and how to treat?. Curr Atheroscler Rep.

[77] Plakogiannis R, Sorbera M, Fischetti B, Chen M (2021). The role of antisense therapies targeting lipoprotein (a). J Cardiovasc Pharmacol.

[78] Ridker PM, Everett BM, Thuren T, MacFadyen JG, Chang WH, Ballantyne C (2017). Antiinflammatory therapy with Canakinumab for atherosclerotic disease. N Engl J Med.

[79] Madjid M, Awan I, Willerson JT, Casscells SW (2004). Leukocyte count and coronary heart disease: implications for risk assessment. J Am Coll Cardiol.

[80] Coller BS (2005). Leukocytosis and ischemic vascular disease morbidity and mortality: is it time to intervene?. Arterioscler Thromb Vasc Biol.

[81] Guasti L, Dentali F, Castiglioni L, Maroni L, Marino F, Squizzato A (2011). Neutrophils and clinical outcomes in patients with acute coronary syndromes and/or cardiac revascularization: a systematic review on more than 34,000 subjects. Thromb Haemost.

[82] Sreejit G, Abdel-Latif A, Athmanathan B, Annabathula R, Dhyani A, Noothi SK (2020). Neutrophil-derived S100A8/A9 amplify granulopoiesis after myocardial infarction. Circulation.

[83] Sreejit G, Nooti SK, Jaggers RM, Athmanathan B, Ho Park K, Al-Sharea A (2022). Retention of the NLRP3 inflammasome-primed neutrophils in the bone marrow is essential for myocardial infarction-induced granulopoiesis. Circulation.

[84] Choi SH, Kim JH, Lim S, Lim JY, Kim KW, Park KS (2017). Monocyte count as a predictor of cardiovascular mortality in older Korean people. Age Ageing.

[85] Tan TP, Arekapudi A, Metha J, Prasad A, Venkatraghavan L (2015). Neutrophil-lymphocyte ratio as predictor of mortality and morbidity in cardiovascular surgery: a systematic review. ANZ J Surg.

[86] Wheeler JG, Mussolino ME, Gillum RF, Danesh J (2004). Associations between differential leucocyte count and incident coronary heart disease: 1764 incident cases from seven prospective studies of 30,374 individuals. Eur Heart J.

[87] Huo Y, Schober A, Forlow SB, Smith DF, Hyman MC, Jung S (2003). Circulating activated platelets exacerbate atherosclerosis in mice deficient in apolipoprotein E. Nat Med.

[88] APT, I. (1994). Collaborative overview of randomised trials of antiplatelet therapy–I: Prevention of death, myocardial infarction, and stroke by prolonged antiplatelet therapy in various categories of patients Antiplatelet Trialists' Collaboration. BMJ.

[89] Lusis AJ (2000). Atherosclerosis Nature.

[90] Dhume AS, Soundararajan K, Hunter WJ, Agrawal DK (2003). Comparison of vascular smooth muscle cell apoptosis and fibrous cap morphology in symptomatic and asymptomatic carotid artery disease. Annals Vasc Surg.

[91] Dhume AS, Agrawal DK (2003). Inability of vascular smooth muscle cells to proceed beyond S phase of cell cycle and increased apoptosis in symptomatic carotid artery disease. J Vasc Surg.

[92] Gould RG (1951). Lipid metabolism and atherosclerosis. Am J Med.

[93] Harrison D, Griendling KK, Landmesser U, Hornig B, Drexler H (2003). Role of oxidative stress in atherosclerosis. Am J Cardiol.

[94] Lusis AJ (2012). Genetics of atherosclerosis. Trends Genet.

[95] Hansson GK, Hermansson A (2011). The immune system in atherosclerosis. Nat Immunol.

[96] Xu S, Pelisek J, Jin ZG (2018). Atherosclerosis Is an Epigenetic Disease. Trends Endocrinol Metab.

[97] Bhatnagar A (2017). Environmental determinants of cardiovascular disease. Circ Res.

[98] Bentzon JF, Otsuka F, Virmani R, Falk E (2014). Mechanisms of plaque formation and rupture. Circ Res.

[99] Libby P (2013). Mechanisms of acute coronary syndromes and their implications for therapy. N Engl J Med.

[100] Rao VH, Rai V, Stoupa S, Subramanian S, Agrawal DK (2016). Tumor necrosis factor-alpha regulates triggering receptor expressed on myeloid cells-1-dependent matrix metalloproteinases in the carotid plaques of symptomatic patients with carotid stenosis. Atherosclerosis.

[101] Battes LC, Cheng JM, Oemrawsingh RM, Boersma E, Garcia-Garcia HM, de Boer SP (2014). Circulating cytokines in relation to the extent and composition of coronary atherosclerosis: results from the Atheroremo-Ivus study. Atherosclerosis.

[102] Virmani R, Kolodgie FD, Burke AP, Farb A, Schwartz SM (2000). Lessons from sudden coronary death: a comprehensive morphological classification scheme for atherosclerotic lesions. Arterioscler Thromb Vasc Biol.

[103] Libby P (2021). The changing landscape of atherosclerosis. Nature.

[104] Wang Y, Meng R, Liu G, Cao C, Chen F, Jin K (2019). Intracranial atherosclerotic disease. Neurobiol Dis.

[105] Sinclair H, Veerasamy M, Bourantas C, Egred M, Nair A, Calvert PA (2016). The role of virtual histology intravascular ultrasound in the identification of coronary artery plaque vulnerability in acute coronary syndromes. Cardiol Rev.

[106] Voros S (2008). Can computed tomography angiography of the coronary arteries characterize atherosclerotic plaque composition? Is the CAT (scan) out of the bag?. JACC Cardiovasc Interv.

[107] Pundziute G, Schuijf JD, Jukema JW, Decramer I, Sarno G, Vanhoenacker PK (2008). Head-to-head comparison of coronary plaque evaluation between multislice computed tomography and intravascular ultrasound radiofrequency data analysis. JACC Cardiovasc Interv.

[108] Hattori K, Ozaki Y, Ismail TF, Okumura M, Naruse H, Kan S (2012). Impact of statin therapy on plaque characteristics as assessed by serial OCT, grayscale and integrated backscatter-IVUS. JACC Cardiovasc Imaging.

[109] Brezinski ME, Harjai KJ (2014). Longitudinal necrotic shafts near TCFAs–a potential novel mechanism for plaque rupture to trigger ACS?. Int J Cardiol.

[110] Farb A, Burke AP, Tang AL, Liang TY, Mannan P, Smialek J (1996). Coronary plaque erosion without rupture into a lipid core: a frequent cause of coronary thrombosis in sudden coronary death. Circulation.

[111] Collet C, Conte E, Mushtaq S, Brouwers S, Shinke T, Coskun AU (2021). Reviewing imaging modalities for the assessment of plaque erosion. Atherosclerosis.

[112] Partida RA, Libby P, Crea F, Jang IK (2018). Plaque erosion: a new in vivo diagnosis and a potential major shift in the management of patients with acute coronary syndromes. Eur Heart J.

[113] Pasterkamp G, den Ruijter HM, Libby P (2017). Temporal shifts in clinical presentation and underlying mechanisms of atherosclerotic disease. Nat Rev Cardiol.

[114] Armstrong ML, Megan MB (1972). Lipid depletion in atheromatous coronary arteries in rhesus monkeys after regression diets. Circ Res.

[115] Rao VH, Rai V, Stoupa S, Subramanian S, Agrawal DK (2016). Data on TREM-1 activation in destabilizing carotid plaques. Data Brief.

[116] Satish M, Agrawal DK (2020). Atherothrombosis and the NLRP3 inflammasome – endogenous mechanisms of inhibition. Transl Res.

[117] Mohindra R, Agrawal DK, Thankam FG (2021). Altered vascular extracellular matrix in the pathogenesis of atherosclerosis. J Cardiovasc Transl Res.

[118] Khwaja B, Thankam FG, Agrawal DK (2021). Mitochondrial DAMPs and altered mitochondrial dynamics in oxLDL-burden in atherosclerosis. Mol Cell Biochem.

[119] Thankam FG, Rai T, Liu J, Tam J, Agrawal DK (2022). Minimally oxidized-LDL-driven alterations in the level of pathological mediators and biological processes in carotid atherosclerosis. Cardiol Cardiovasc Med.

[120] Patel P, Rai V, Agrawal DK (2023). Role of oncostatin-M in ECM remodeling and plaque vulnerability. Mol Cell Biochem.

[121] Honda K, Matoba T, Antoku Y, Koga JI, Ichi I, Nakano K (2018). Lipid-Lowering therapy with ezetimibe decreases spontaneous atherothrombotic occlusions in a rabbit model of plaque erosion: a role of serum oxysterols. Arterioscler Thromb Vasc Biol.

[122] Aikawa M, Rabkin E, Sugiyama S, Voglic SJ, Fukumoto Y, Furukawa Y (2001). An HMG-CoA reductase inhibitor, cerivastatin, suppresses growth of macrophages expressing matrix metalloproteinases and tissue factor in vivo and in vitro. Circulation.

[123] Hu S, Yonetsu T, Jia H, Karanasos A, Aguirre AD, Tian J (2014). Residual thrombus pattern in patients with ST-segment elevation myocardial infarction caused by plaque erosion versus plaque rupture after successful fibrinolysis: an optical coherence tomography study. J Am Coll Cardiol.

[124] Azevedo A, Prado AF, Antonio RC, Issa JP, Gerlach RF (2014). Matrix metalloproteinases are involved in cardiovascular diseases. Basic Clin Pharmacol Toxicol.

[125] Papazafiropoulou A, Tentolouris N (2009). Matrix metalloproteinases and cardiovascular diseases. Hippokratia.

[126] Rajavashisth TB, Liao JK, Galis ZS, Tripathi S, Laufs U, Tripathi J (1999). Inflammatory cytokines and oxidized low density lipoproteins increase endothelial cell expression of membrane type 1-matrix metalloproteinase. J Biol Chem.

[127] d'Ortho MP, Will H, Atkinson S, Butler G, Messent A, Gavrilovic J (1997). Membrane-type matrix metalloproteinases 1 and 2 exhibit broad-spectrum proteolytic capacities comparable to many matrix metalloproteinases. Eur J Biochem.

[128] Sugiyama S, Kugiyama K, Aikawa M, Nakamura S, Ogawa H, Libby P (2004). Hypochlorous acid, a macrophage product, induces endothelial apoptosis and tissue factor expression: involvement of myeloperoxidase-mediated oxidant in plaque erosion and thrombogenesis. Arterioscler Thromb Vasc Biol.

[129] Tricot O, Mallat Z, Heymes C, Belmin J, Leseche G, Tedgui A (2000). Relation between endothelial cell apoptosis and blood flow direction in human atherosclerotic plaques. Circulation.

[130] Mullick AE, Soldau K, Kiosses WB, Bell TA, Tobias PS, Curtiss LK (2008). Increased endothelial expression of Toll-like receptor 2 at sites of disturbed blood flow exacerbates early atherogenic events. J Exp Med.

[131] Franck G, Mawson T, Sausen G, Salinas M, Masson GS, Cole A (2017). Flow perturbation mediates neutrophil recruitment and potentiates endothelial injury via TLR2 in mice: implications for superficial erosion. Circ Res.

[132] Kolodgie FD, Burke AP, Farb A, Weber DK, Kutys R, Wight TN (2002). Differential accumulation of proteoglycans and hyaluronan in culprit lesions: insights into plaque erosion. Arterioscler Thromb Vasc Biol.

[133] Kolodgie FD, Burke AP, Wight TN, Virmani R (2004). The accumulation of specific types of proteoglycans in eroded plaques: a role in coronary thrombosis in the absence of rupture. Curr Opin Lipidol.

[134] Parisi LR, Morrow LM, Visser MB, Atilla-Gokcumen GE (2018). Turning the spotlight on lipids in non-apoptotic cell death. ACS Chem Biol.

[135] Quillard T, Araujo HA, Franck G, Shvartz E, Sukhova G, Libby P (2015). TLR2 and neutrophils potentiate endothelial stress, apoptosis and detachment: implications for superficial erosion. Eur Heart J.

[136] Jia H, Dai J, Hou J, Xing L, Ma L, Liu H (2017). Effective anti-thrombotic therapy without stenting: intravascular optical coherence tomography-based management in plaque erosion (the EROSION study). Eur Heart J.

[137] Burke AP, Kolodgie FD, Farb A, Weber DK, Malcom GT, Smialek J (2001). Healed plaque ruptures and sudden coronary death: evidence that subclinical rupture has a role in plaque progression. Circulation.

[138] Rioufol G, Gilard M, Finet G, Ginon I, Boschat J, Andre-Fouet X (2004). Evolution of spontaneous atherosclerotic plaque rupture with medical therapy: long-term follow-up with intravascular ultrasound. Circulation.

[139] Russo M, Fracassi F, Kurihara O, Kim HO, Thondapu V, Araki M (2020). Healed plaques in patients with stable angina pectoris. Arterioscler Thromb Vasc Biol.

[140] Teng Z, Degnan AJ, Sadat U, Wang F, Young VE, Graves MJ (2011). Characterization of healing following atherosclerotic carotid plaque rupture in acutely symptomatic patients: an exploratory study using in vivo cardiovascular magnetic resonance. J Cardiovasc Magn Reson.

[141] Wang C, Hu S, Wu J, Yu H, Pan W, Qin Y (2019). Characteristics and significance of healed plaques in patients with acute coronary syndrome and stable angina: an in vivo OCT and IVUS study. EuroIntervention.

[142] Fracassi F, Crea F, Sugiyama T, Yamamoto E, Uemura S, Vergallo R (2019). Healed culprit plaques in patients with acute coronary syndromes. J Am Coll Cardiol.

[143] Shimokado A, Matsuo Y, Kubo T, Nishiguchi T, Taruya A, Teraguchi I (2018). In vivo optical coherence tomography imaging and histopathology of healed coronary plaques. Atherosclerosis.

[144] Kubo T, Maehara A, Mintz GS, Doi H, Tsujita K, Choi SY (2010). The dynamic nature of coronary artery lesion morphology assessed by serial virtual histology intravascular ultrasound tissue characterization. J Am Coll Cardiol.

[145] Yamamoto MH, Yamashita K, Matsumura M, Fujino A, Ishida M, Ebara S (2017). Serial 3-vessel optical coherence tomography and intravascular ultrasound analysis of changing morphologies associated with lesion progression in patients with stable angina pectoris. Circ Cardiovasc Imaging.

[146] Phipps JE, Vela D, Hoyt T, Halaney DL, Mancuso JJ, Buja LM (2015). Macrophages and intravascular OCT bright spots: a quantitative study. JACC Cardiovasc Imaging.

[147] Raber L, Koskinas KC, Yamaji K, Taniwaki M, Roffi M, Holmvang L (2019). Changes in coronary plaque composition in patients With acute myocardial infarction treated with high-intensity statin Therapy (IBIS-4): a serial optical coherence tomography study. JACC Cardiovasc Imaging.

[148] Hougaard M, Hansen HS, Thayssen P, Maehara A, Antonsen L, Junker A (2020). Influence of ezetimibe on plaque morphology in patients with ST elevation myocardial infarction assessed by optical coherence tomography: an Octivus sub-study. Cardiovasc Revasc Med.

[149] Vergallo R, Crea F (2020). Atherosclerotic plaque healing. N Engl J Med.

[150] Singh D, Rai V, Agrawal DK (2022). Non-coding RNAs in regulating plaque progression and remodeling of extracellular matrix in atherosclerosis. Int J Med Sci.

